# Imaging of Acute Invasive Fungal Rhinosinusitis in a Patient with Gorlin Syndrome and Acute Lymphocytic Leukemia

**DOI:** 10.1155/2013/272314

**Published:** 2013-06-18

**Authors:** S. T. Donovan, J. W. Thompson, J. T. Sandlund, E. E. Adderson, E. K. Pivnick, J. H. Harreld

**Affiliations:** ^1^Department of Otolaryngology, University of Florida College of Medicine, 1600 SW Archer Road, Gainesville, FL 32608, USA; ^2^Department of Otolaryngology, University of Tennessee Health Sciences Center, 910 Madison Avenue, Memphis, TN 38163, USA; ^3^Department of Oncology, St. Jude Children's Research Hospital, 262 Danny Thomas Boulevard, MS-260, Memphis, TN 38105, USA; ^4^Department of Infectious Diseases, St. Jude Children's Research Hospital, 262 Danny Thomas Boulevard, MS-320, Memphis, TN 38105, USA; ^5^Departments of Pediatrics and Ophthalmology, University of Tennessee Health Sciences Center, 711 Jefferson Avenue, Suite 523, Memphis, TN 38105, USA; ^6^Department of Radiological Sciences, St. Jude Children's Research Hospital, 262 Danny Thomas Boulevard, MS-220, Memphis, TN 38105, USA

## Abstract

Gorlin Syndrome (GS), also known as nevoid basal cell carcinoma syndrome, is a rare autosomal dominant condition characterized by developmental abnormalities and predisposition to certain neoplasms. Acute invasive fungal rhinosinusitis (AIFRS) is an uncommon clinical entity characterized by high morbidity and mortality. In immunocompromised patients, computed tomography plays a critical role in screening for suspected AIFRS. However, due to the association between exposure to ionizing radiation and subsequent development of malignancies in patients with GS, patients with GS and suspected AIFRS present a unique and challenging clinical scenario. We present a case of a pediatric patient with GS and acute lymphocytic leukemia (ALL) diagnosed with AIFRS; to the best of our knowledge, it is the only case described in the literature.

## 1. Introduction

Acute invasive fungal rhinosinusitis (AIFRS) is a serious condition characterized by high morbidity and a high mortality rate of 50%–80% in immunocompromised patients [1]. Due to the need for expeditious imaging in suspected cases to assist in diagnosis and management, CT is often used as the initial diagnostic modality, though magnetic resonance imaging (MRI) plays a critical role in the diagnosis of AIFRS [2].

Gorlin Syndrome (GS) is a rare autosomal dominant condition characterized by multiple basal cell carcinomas of the skin, odontogenic keratocysts, dermoid cysts, mesenteric cysts, pits of the palms and soles, eye anomalies, variable developmental delay, and skeletal abnormalities including macrocephaly, tall stature, and rib and vertebral anomalies [3, 4]. Gorlin Syndrome is due to a mutation in PTCH1, a tumor suppressor gene involved in the Sonic Hedgehog ligand-signaling pathway that plays a role in normal embryonic development [4]. Loss of heterozygosity due to this mutation predisposes patients to the characteristic features of the syndrome, as well as medulloblastomas, rhabdomyosarcomas and other cancers [4, 5]. Because exposure to ionizing radiation is an important cofactor for secondary malignancies in patients with GS, CT and X-ray should be used judiciously in these patients [6]. Thus, screening as well as diagnostic testing for fungal sinus disease presents a particular challenge in these patients. 

We present a case of a pediatric patient with GS and ALL diagnosed with AIFRS. We discuss the special considerations of this case, in addition to the characteristics of Gorlin Syndrome as they apply to the otolaryngologist, the clinical and radiographic findings of AIFRS, and role of imaging in the diagnosis of AIFRS.

## 2. Case Report

An 8-year-old Caucasian male presented to our facility with a 2-week history of refractory clear rhinorrhea, facial pain, jaw pain, and somnolence. The patient had previously been diagnosed with sporadic GS after being found to have multiple basal cell nevi and pitting of the palms and soles at the age of 5, as well as more recently a second relapse of ALL and chemotherapy-associated neutropenia (absolute neutrophil count 0/mm^3^). Family history was negative for GS but revealed a maternal third cousin with osteosarcoma and a maternal second cousin with ALL. The patient denied double vision, decreased vision, and nasal obstruction but did have one episode of epistaxis on the day of admission. There was no known exposure to nickel or significant secondhand smoke exposure. At the time of evaluation he was receiving micafungin for anti-fungal prophylaxis. On physical examination there was a pale gray lesion on the palate suspicious for avascular tissue, versus underlying Gorlin-related cyst. Black debris in the nares was believed to be due to epistaxis; deeper examination revealed grey/brown tissue and nasal polyposis. Because of concerns regarding ionizing radiation, evaluation with MRI was pursued.

**Figure 1:**
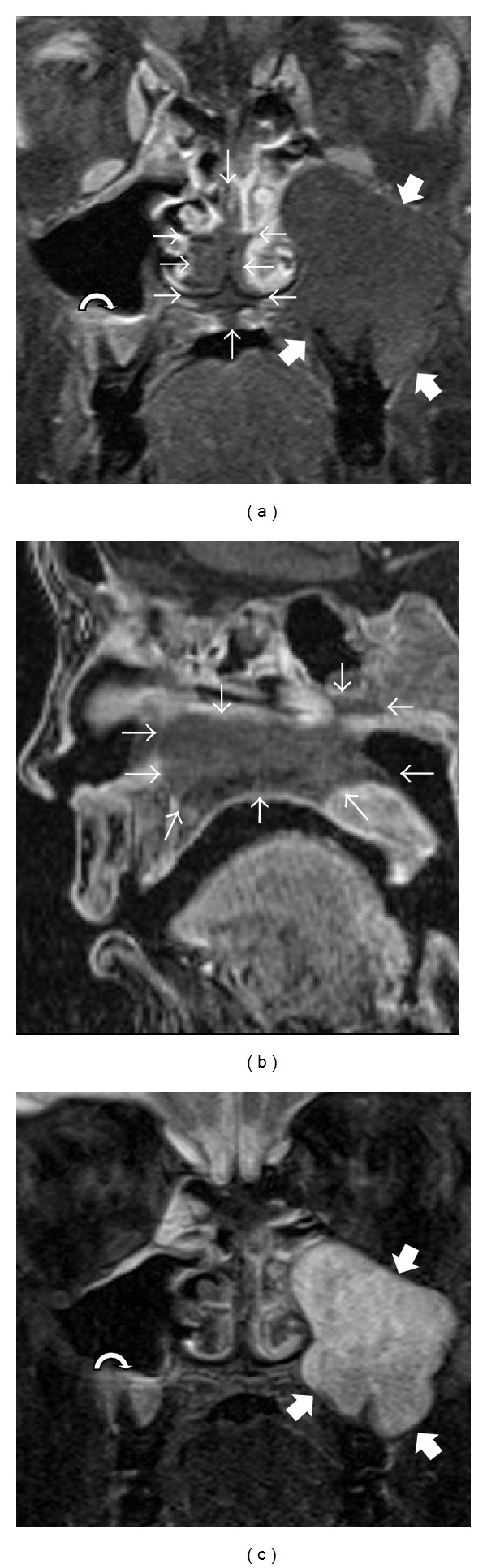
(a) Coronal postcontrast fat-saturated T1-weighted images (T1WI) and (b) sagittal postcontrast T1WI demonstrate sharply delineated hypoenhancement of the nasal septum and bilateral inferior turbinates extending inferiorly to the hard palate and superiorly to the inferior aspect of the sphenoid body (outlined by small arrows) without significant associated mucosal edema on STIR images (c). An odontogenic cyst centered on the crown of a left maxillary molar fills and expands the left maxillary sinus (thick arrows). A smaller maxillary odontogenic cyst is present on the right (curved arrow).

MRI demonstrated sharply delineated hypoenhancement of the nasal septum and bilateral inferior turbinates consistent with necrosis, which extended inferiorly to the hard palate and superiorly to the inferior aspect of the sphenoid body (Figures 1(a) and 1(b)). There was no MRI evidence of intracranial extension. There was no significant mucosal swelling or edema, or other mucosal signal abnormalities, on short-tau inversion recovery (STIR) images (Figure 1(c)). A large cyst centered on the crown of a left maxillary molar consistent with odontogenic cyst extended into and expanded the left maxillary sinus (Figure 1). Smaller cysts with a similar appearance surrounded the roots of a right maxillary molar (Figures 1(a) and 1(b)) and a mandibular molar (not shown). A right convexity collection consistent with subdural hemorrhage complicated the imaging findings.

Treatment with liposomal amphotericin B and oral posaconazole was initiated and micafungin continued. Surgical biopsy taken from the hard palate revealed focal areas of necrosis. Gömöri methenamine-silver stains demonstrated tissue invasion by wide, sparsely septated hyphae, consistent with *Zygomycetes* or *Aspergillus*. Tissue cultures were sterile. Due to the poor prognosis of the patient's underlying disease and quality of life concerns, aggressive surgical management was not pursued.

Despite antifungal therapy, the patient continued to deteriorate clinically. CT at one month after diagnosis demonstrated extension of sinonasal disease, new orbital invasion, demineralization of the nasal turbinates, and a small focus of nasal septal destruction. The patient's course was complicated by *Enterococcus faecium* bacteremia, disseminated *Candida famata* infection, and progressive obstruction of the upper airway. He expired two months following the diagnosis of AIFRS.

## 3. Discussion

In GS the predisposition to certain solid tumors including medulloblastoma and rhabdomyosarcoma due to genetic defects in the Sonic Hedgehog pathway has been well described [5]. An association between GS and hematologic malignancies such as acute lymphocytic leukemia, as occurred in our patient, to our knowledge has not been previously described [5]. Alternatively, our patient may have developed ALL due to another cancer predisposing gene as the family history reveals two maternal relatives with osteosarcoma and ALL, though this theory was unconfirmed due to unavailability of the affected individuals and their medical records. There was no first degree relative with cancer or malignancy.

At our institution, CT is frequently utilized to screen immunocompromised patients with ALL for sinus disease, particularly prior to bone marrow transplant. This diagnostic method becomes problematic, however, in patients with GS, who are particularly susceptible to ionizing radiation [6]. Treatment-dose ionizing radiation typically results in the formation of advanced and difficult-to-treat basal cell carcinomas three to six months later [7]. For this reason, CT and X-ray, which use ionizing radiation, are relatively contraindicated in patients with GS and should be used judiciously.

Early recognition and diagnosis of GS will guide appropriate treatment for patients, including minimizing of both therapeutic and diagnostic radiation [8]. While odontogenic keratocysts and basal cell carcinomas aid in the diagnosis of GS, these characteristics are usually progressive in nature and may appear in later childhood and adolescence. Thus, awareness of features presenting earlier in life, such as dermoid cysts, which may be recognizable in infancy, may raise clinical suspicion and be helpful in establishing an early diagnosis. Other craniofacial clues include relative macrocephaly and hypertelorism (prevalence of each approaching nearly 50%) and frontal bossing (present in over 25% of patients) [6]. Additionally, there is an association with cleft lip and palate [6]. Radiographically, the most common finding is calcification of the falx cerebri, which typically takes years to develop but may be present earlier in life. Other radiographic clues include bridging of the sella turcica (seen in over 66% of patients) and abnormal frontal sinus aeration [6]. Significantly, medulloblastoma occurs earlier in life in patients with GS than in the general population; it has been suggested that over 4% of patients with medulloblastoma younger than the age of 5 may have undiagnosed GS and that younger patients with medulloblastoma, particularly of the desmoplastic variant, should be screened clinically for GS [7].

Acute invasive fungal rhinosinusitis (AIFRS), an uncommon entity, occurs almost exclusively in patients who are immunosuppressed or those with diabetes mellitus. Patients can present with serosanguinous rhinorrhea, nasal fullness or obstruction, sinus pain, diplopia, loss of visual acuity, cranial nerve involvement, or with nonspecific signs such as fever [1, 9]. Orbital involvement is typical, although it was not present until late in this case [10]. Bony destruction is common [11]. With an overall mortality rate of 50%–80%, prompt diagnosis and management are key [1]. The combination of prompt surgical intervention and antifungal therapy has proved superior to monotherapy with intravenous antifungals [11]. 

Previously, conventional wisdom dictated that CT was sufficient for radiographic evaluation for AIFRS. However, the sensitivity of MRI in detecting early findings of AIFRS, such as periantral fat infiltration and tissue necrosis, is superior to that of CT; [2, 12] bony destruction detectable by CT is a late finding with respect to disease progression. MRI is therefore helpful in differentiating AIFRS from other inflammatory entities and can make a crucial difference in patient care. MRI is particularly suitable in circumstances of high clinical suspicion for early or subtle disease, or relative contraindication to CT, both present in this case.

Hypoenhancement and near-normal T2 signal in invasive fungal disease due to microvascular invasion of hyphae and subsequent necrosis in the absence of an immune response sufficient to cause substantial mucosal edema, as seen in this case, are suggestive of AIFRS; this is distinct from the diffuse enhancement and T2 hyperintensity typically seen in other inflammatory diseases [12, 13]. A pattern of nasal cavity, orbital, ethmoid, and maxillary involvement is highly suggestive [14]. For the head and neck surgeon, MRI can better delineate areas of involvement, which may guide surgical intervention in the form of endoscopic versus open surgical debridement and simple debridement versus more extensive resections [15].

This report of AIFRS in a patient with GS represents an uncommonly encountered comorbidity and exhibits a unique and challenging clinical scenario. Because ionizing radiation should be minimized in patients with GS, in this clinical situation, MRI should be promptly performed for timely and accurate diagnosis of AIFRS.

## References

[1] Gillespie MB, O’Malley BW, Francis HW (1998). An approach to fulminant invasive fungal rhinosinusitis in the immunocompromised host. *Archives of Otolaryngology*.

[2] Groppo ER, El-Sayed IH, Aiken AH, Glastonbury CM (2011). Computed tomography and magnetic resonance imaging characteristics of acute invasive fungal sinusitis. *Archives of Otolaryngology*.

[3] Gorlin RJ, Goltz RW (1960). Multiple nevoid basal-cell epithelioma, jaw cysts and bifid rib: a syndrome. *The New England journal of medicine*.

[4] Muzio LL (2008). Nevoid basal cell carcinoma syndrome (Gorlin syndrome). *Orphanet Journal of Rare Diseases*.

[5] Cajaiba MM, Bale AE, Alvarez-Franco M, McNamara J, Reyes-Múgica M (2006). Rhabdomyosarcoma, Wilms tumor, and deletion of the patched gene in Gorlin syndrome. *Nature Clinical Practice Oncology*.

[6] Kimonis VE, Goldstein AM, Pastakia B (1997). Clinical manifestations in 105 persons with nevoid basal cell carcinoma syndrome. *The American Journal of Medical Genetics*.

[7] Evans DGR, Farndon LA, Burnell LD, Rao Gattamaneni H, Birch JM (1991). The incidence of Gorlin syndrome in 173 consecutive cases of medulloblastoma. *British Journal of Cancer*.

[8] Bree AF, Shah MR (2011). Consensus statement from the first international colloquium on basal cell nevus syndrome (BCNS). *The American Journal of Medical Genetics A*.

[9] Abbasi S, Shenep JL, Hughes WT, Flynn PM (1999). Aspergillosis in children with cancer: a 34-year experience. *Clinical Infectious Diseases*.

[10] Chandrasekharan R, Thomas M, Rupa V (2012). Comparative study of orbital involvement in invasive and non-invasive fungal sinusitis. *Journal of Laryngology and Otology*.

[11] Kasapoglu F, Coskun H, Ozmen OA, Akalin H, Ener B (2010). Acute invasive fungal rhinosinusitis: evaluation of 26 patients treated with endonasal or open surgical procedures. *Otolaryngology*.

[12] Safder S, Carpenter JS, Roberts TD, Bailey N (2010). The “black turbinate” sign: an early MR imaging finding of nasal mucormycosis. *The American Journal of Neuroradiology*.

[13] Rassi SJ, Melkane AE, Rizk HG, Dahoui HA (2009). Sinonasal mucormycosis in immunocompromised pediatric patients. *Journal of Pediatric Hematology/Oncology*.

[14] Herrera DA, Dublin AB, Ormsby EL, Aminpour S, Howell LP (2009). Imaging findings of rhinocerebral mucormycosis. *Skull Base*.

[15] Howells RC, Ramadan HH (2001). Usefulness of computed tomography and magnetic resonance in fulminant invasive fungal rhinosinusitis. *The American Journal of Rhinology*.

